# Comprehensive Multi-Omics Characterization of CYP2R1 as a Diagnostic and Functional Biomarker in Hepatocellular Carcinoma

**DOI:** 10.3390/medsci14020178

**Published:** 2026-04-01

**Authors:** Hyun Sun Jung, Geum Ok Baek, Moon Gyeong Yoon, Se Ha Jang, Jae Youn Cheong, Soon Sun Kim, Jung Woo Eun

**Affiliations:** 1 Department of Biomedical Sciences, Ajou University Graduate School of Medicine, 164 Worldcup-ro, Yeongtong-gu, Suwon 16499, Republic of Korea; 2 Department of Gastroenterology, Ajou University School of Medicine, 164 Worldcup-ro, Yeongtong-gu, Suwon 16499, Republic of Koreajaeyoun620@gmail.com (J.Y.C.);; 3 Department of Medical Affairs, Hugel Inc., Gangnam-daero 133, Gangnam-gu, Seoul 06068, Republic of Korea

**Keywords:** *CYP2R1*, cytochrome P450, diagnosis, hepatocellular carcinoma, prognostic biomarker

## Abstract

**Background:** Hepatocellular carcinoma (HCC) remains a major cause of cancer mortality worldwide, yet reliable molecular biomarkers for early diagnosis and prognosis are limited. The cytochrome P450 (CYP) enzyme superfamily plays central roles in hepatic metabolism and tumor biology, but its global dysregulation in HCC has not been comprehensively defined. **Methods:** Here, we systematically analyzed 57 CYP genes across multi-cohort transcriptomic datasets (GepLiver, TCGA-LIHC, HCCDB2.0) to delineate their diagnostic, prognostic, and functional significance. **Results:** *CYP2R1* emerged as a consistently upregulated and clinically relevant member, showing excellent diagnostic accuracy (AUC = 0.95, 95% CI: 0.94–0.98, *p* < 0.001) and strong overexpression validated across independent cohorts and spatial transcriptomics. Prognostic modeling identified *CYP2C9* (favorable) and *CYP26B1* (unfavorable) as independent survival markers. Functional enrichment analyses revealed that high *CYP2R1* expression was associated with activation of DNA repair and replication pathways (NES = 1.31, adjusted *p* = 1.05 × 10^−5^) and with co-expression of core repair genes such as *POLA2*, *LIG1*, and *PCNA*. Moreover, *CYP2R1* correlated with myeloid-derived suppressor cell infiltration, suggesting an immunosuppressive phenotype. **Conclusions:** These findings establish *CYP2R1* as a novel metabolic and immunogenomic biomarker in HCC, linking hepatic metabolism, genomic maintenance, and tumor immune modulation.

## 1. Introduction

Hepatocellular carcinoma (HCC) is a major global health burden and the leading cause of primary liver cancer, accounting for more than 800,000 deaths annually [1]. Early detection and improved risk stratification remain pressing unmet needs, as most HCC cases are diagnosed at advanced stages when therapeutic options are limited and prognosis is poor [2,3]. Despite progress in surveillance and molecular therapy, the heterogeneity of HCC driven by diverse etiologies such as viral hepatitis, alcohol, and metabolic dysfunction–associated steatotic liver disease (MASLD) continues to complicate biomarker discovery and clinical management [4].

The cytochrome P450 (CYP) enzyme family consists of 57 functional genes in humans and catalyzes the oxidative metabolism of numerous endogenous and exogenous substrates, including drugs, toxins, fatty acids, steroids, and vitamins [5]. Highly expressed in the liver, CYPs are central to xenobiotic detoxification, metabolic homeostasis, and the biosynthesis of bile acids and cholesterol [6,7]. The CYP2, CYP3, and CYP4 clusters account for the majority of phase I metabolic reactions [5,8].

Altered CYP expression significantly affects liver disease progression, carcinogen metabolism, and drug response [6,9]. Transcriptomic studies show that major hepatic CYPs, including *CYP1A2*, *CYP2C8*, *CYP2C9*, *CYP2E1*, and *CYP3A4*, are downregulated in HCC and have been suggested as potential biomarkers or predictors of therapeutic response, although findings remain inconsistent [10]. Other CYP members involved in lipid and bile acid metabolism, such as *CYP4A11*, *CYP8B1*, *CYP7A1*, and *CYP26B1*, are closely linked to hepatocarcinogenesis, but their roles in HCC remain poorly understood [5,11].

Despite the increasing availability of large-scale datasets such as TCGA and GepLiver, prior analyses have typically focused on isolated CYP genes rather than the entire family, leaving the comprehensive landscape of CYP dysregulation in HCC largely undefined. An integrative, multi-cohort approach that spans transcriptomic, proteomic, and clinical layers is thus essential to delineate the roles of CYPs in liver tumor biology and to identify novel molecular determinants of disease behavior.

Among the CYP family, *CYP2R1* has recently attracted attention as a key vitamin D 25-hydroxylase involved in systemic vitamin D activation [12], and its crystal structure in complex with vitamin D3 has been resolved, providing mechanistic insight into substrate recognition and catalysis [13]. Given the emerging evidence linking vitamin D signaling to cancer metabolism, immune regulation, and genomic stability [14,15], aberrant *CYP2R1* expression may represent a crucial metabolic alteration in HCC. However, its role in liver cancer has not been systematically characterized.

In this study, we performed a comprehensive multi-omics investigation of all 57 CYP genes in HCC, integrating analyses from GepLiver, TCGA-LIHC, and HCCDB2.0 cohorts. Through rigorous screening and cross-validation, we identified *CYP2R1* as a consistently upregulated and clinically relevant biomarker. We further evaluated its diagnostic and prognostic significance, characterized its associated biological pathways, including DNA repair activation and immune modulation, and validated its expression using independent clinical and spatial transcriptomic datasets. Collectively, our findings establish *CYP2R1* as a novel metabolic and immunogenomic biomarker in HCC, providing new insight into the intersection between hepatic metabolism, genomic maintenance, and tumor immune microenvironment.

## 2. Materials and Methods

### 2.1. Data Collection and Preprocessing

Publicly available transcriptomic and clinical datasets were retrieved from The Cancer Genome Atlas Liver Hepatocellular Carcinoma (TCGA-LIHC; https://portal.gdc.cancer.gov/ (accessed on 19 February 2026)), GepLiver (http://www.gepliver.org (accessed on 19 February 2026)), and HCCDB2.0 (http://lifeome.net/database/hccdb/home.html (accessed on 19 February 2026)). Gene-level normalized expression matrices (log_2_(TPM + 1)) were used for all analyses. Sample metadata were harmonized by disease category, and only primary HCC and matched non-tumor liver tissues were included. Batch correction and normalization were performed using the R package limma (v3.58.0). For protein-level evaluation, immunohistochemistry (IHC) images and expression scores were obtained from the Human Protein Atlas (HPA; https://www.proteinatlas.org/ (accessed on 19 February 2026)).

### 2.2. Oncoprint and Mutation Landscape Analysis

Mutation status, copy number variation, and mRNA expression alterations of all 57 CYP genes were analyzed using the cBioPortal for Cancer Genomics (https://www.cbioportal.org/ (accessed on 19 February 2026)). Data from the TCGA-LIHC cohort (n = 348) were used to construct Oncoprints and frequency plots. Integrated mutation types and alteration frequencies were visualized to assess genomic variation patterns and their relative prevalence among HCC patients.

### 2.3. Differential Expression and Correlation Analysis

All statistical analyses were performed using R (version 4.5.1; R Foundation for Statistical Computing, Vienna, Austria). Differential expression between HCC and non-tumor tissues was computed using the limma package (version 3.64.3), applying an adjusted *p* < 0.05 (Benjamini–Hochberg correction) and |log_2_FC| > 1 as the threshold for significance. Volcano plots and hierarchical heatmaps were generated using ggplot2 (version 3.5.2) and ComplexHeatmap (version 2.24.1) packages. Pairwise correlations among CYP genes were calculated using Pearson’s correlation coefficient with multiple-testing correction (false discovery rate < 0.05).

### 2.4. Diagnostic and Prognostic Evaluation

Diagnostic performance was assessed via receiver operating characteristic (ROC) curve analysis using the GraphPad Prism (v10.6.1; GraphPad Software, San Diego, CA, USA). Area under the curve (AUC) values with 95% confidence intervals were calculated, and the DeLong test was used for statistical comparison. For diagnostic screening, receiver operating characteristic (ROC) curves were first generated in the TCGA-LIHC cohort to assess the ability of each candidate CYP gene to discriminate HCC (n = 371) from non-tumor liver tissues (n = 50). TCGA-LIHC served as the discovery cohort to prioritize the most discriminatory CYP markers, including *CYP2R1*, based on univariable discrimination. The diagnostic performance of *CYP2R1* was subsequently evaluated in two independent cohorts (GepLiver and the Ajou University surgical cohort) using the same ROC framework. In the GepLiver cohort, two clinically relevant diagnostic comparisons were performed: (i) HCC versus all non-tumor liver tissues (including normal liver and chronic liver diseases) and (ii) HCC versus chronic liver disease tissues only, excluding normal controls. Prognostic analysis was performed using univariate and multivariate Cox proportional hazards regression models with the survival package. For overall survival analysis in TCGA-LIHC, 343 patients with complete data and 119 death events were included. Variables that reached *p* < 0.05 in univariate analysis were entered into the multivariable Cox regression model. In total, 21 variables were evaluated in the multivariable analysis, corresponding to an events-per-variable ratio of approximately 5.7, which is considered acceptable for exploratory prognostic analyses aimed at identifying candidate biomarkers rather than constructing a fully optimized prediction model. Variables with *p* < 0.05 in univariate analysis were included in the multivariate model. Kaplan–Meier survival curves were generated and compared using the log-rank test.

### 2.5. Clinical Samples and Ethical Approval

Paired tumor and adjacent non-tumor liver tissues were obtained from 110 patients who underwent surgical resection for HCC at Ajou University Hospital (Suwon, Republic of Korea). Clinical characteristics and sample information were retrieved from medical records and the Ajou Human Source Bank.

Demographic and clinical variables collected for the cohort included age, sex, disease etiology (e.g., hepatitis B virus, hepatitis C virus, alcohol-related liver disease, or metabolic-associated liver disease), body mass index (BMI), alcohol consumption history, smoking status, cirrhosis status, and tumor stage according to both the Barcelona Clinic Liver Cancer (BCLC) classification and the pathological UICC staging system. These clinical characteristics of the validation cohort are summarized in Supplementary Table S6.

All tumor and adjacent non-tumor liver tissues were collected from the same patients at the time of surgical resection and processed under a standardized institutional protocol. The paired design enables intra-individual comparison and reduces potential confounding related to demographic factors, medication exposure, metabolic conditions, and alcohol use.

In a subset of patients, longitudinal paired samples consisting of primary tumor (PT) and recurrent tumor (RT) tissues were available. For exploratory analysis, *CYP2R1* expression was quantified in these paired PT–RT samples using qRT-PCR, allowing within-patient comparison of expression changes during tumor recurrence.

In addition, plasma samples were obtained from individuals with normal liver (n = 15), chronic hepatitis (n = 10), metabolic-associated steatohepatitis (MASH, n = 8), liver cirrhosis (n = 21), and hepatocellular carcinoma (HCC, n = 88) through the Ajou Human Source Bank under the same institutional review board approvals. These samples were used for exploratory ELISA-based measurement of circulating *CYP2R1*. All experiments were performed in accordance with the Declaration of Helsinki, and the study was approved by the Institutional Review Board of Ajou University Hospital (AJOUIRB-EX-2022-389 and AJOUIRB-EX-2024-332, date of approval: 4 October 2022), each corresponding to distinct sample and data collection periods. Anonymous samples and clinical data were provided by the Ajou Human Source Bank, and the requirement for informed consent was waived.

### 2.6. RNA Extraction and Quantitative Reverse Transcription PCR

Total RNA was isolated from liver tissues using TRIzol reagent (Invitrogen, Carlsbad, CA, USA) or the RNeasy Mini Kit (Qiagen, Hilden, Germany). cDNA was synthesized using the PrimeScript™ RT Master Mix (TaKaRa Bio, Otsu, Japan). qRT-PCR was carried out using AmfiSure qGreen Q-PCR Master Mix (GenDEPOT, Barker, TX, USA) on either the Applied Biosystems 7300 Real-Time PCR System (Applied Biosystems, Foster City, CA, USA) or the Bio-Rad CFX Connect Real-Time PCR Detection System (Bio-Rad Laboratories, Hercules, CA, USA). Expression levels were normalized to *HMBS* as an internal reference gene. The primer (Integrated DNA Technologies, Coralville, IA, USA) sequences used were as follows: *HMBS* (NM_000190.3), forward 5′-ACG GCT CAG ATA GCA TAC AAG AG-3′ and reverse 5′-GTT ACG AGC AGT GAT GCC TAC C-3′; and *CYP2R1* (NM_001004065.1), forward 5′-GAG GCA TAT CAA CTG TGG TTC T-3′ and reverse 5′-TGG AAT TGA GTA AGC CTC CCA TT-3′. qRT-PCR experiments were performed with at least three technical replicates for each sample, and relative expression levels were calculated using the 2^−ΔΔCt^ method after normalization to the housekeeping gene.

### 2.7. Enzyme-Linked Immunosorbent Assay (ELISA)

Plasma CYP2R1 concentrations were measured using a commercially available ELISA kit (Cusabio, Wuhan, China) according to the manufacturer’s instructions. Diagnostic performance for distinguishing HCC from non-tumor liver disease controls was evaluated using receiver operating characteristic (ROC) analysis.

### 2.8. Functional Enrichment and Gene Set Enrichment Analysis

Genes significantly correlated with *CYP2R1* expression (|r| ≥ 0.2, adjusted *p* < 0.05) were subjected to pathway enrichment using EnrichR (https://maayanlab.cloud/Enrichr/ (accessed on 19 February 2026)) across the Reactome Pathways 2024, KEGG 2021 Human, MSigDB Hallmark 2020, and WikiPathways 2024 Human databases. Gene Set Enrichment Analysis (GSEA) was performed using the clusterProfiler package (v4.8.3) with the Hallmark gene sets (h.all.v2025.1.Hs.symbols.gmt). Ranked gene lists based on log_2_FC from *CYP2R1*-high (Q1) vs. *CYP2R1*-low (Q4) tumors were used as input. Normalized enrichment scores (NES) and adjusted *p*-values were computed based on 1000 permutations, and results with FDR < 0.25 were considered significant.

### 2.9. Immune Infiltration Analysis

Immune cell infiltration analysis was conducted using the TIMER2.0 platform (http://timer.cistrome.org/ (accessed on 10 June 2025)). TCGA-LIHC RNA-seq data were analyzed using the TIMER deconvolution algorithm implemented in TIMER2.0 to estimate immune cell abundance from bulk transcriptomic profiles. Correlations between *CYP2R1* expression and immune infiltration levels were evaluated using Spearman’s correlation with tumor purity adjustment as implemented in the TIMER2.0 platform.

### 2.10. Statistical Analysis

All statistical analyses were performed using R software (v4.3.2) and GraphPad Prism. Group comparisons were performed using Welch’s *t*-test (two groups) or one-way ANOVA followed by Tukey’s post hoc test (multiple groups). Correlation coefficients were calculated using Pearson’s or Spearman’s method as appropriate, with *p*-values adjusted using the Benjamini–Hochberg procedure. Data are presented as mean ± standard deviation (SD) or mean ± standard error of the mean (SEM), as indicated in the figure legends. All statistical tests were two-sided, and differences were considered statistically significant at *p* < 0.05 or adjusted *p* < 0.05 unless otherwise stated.

## 3. Results

### 3.1. Heterogeneous Expression of CYP Genes in HCC

**Figure 1:**
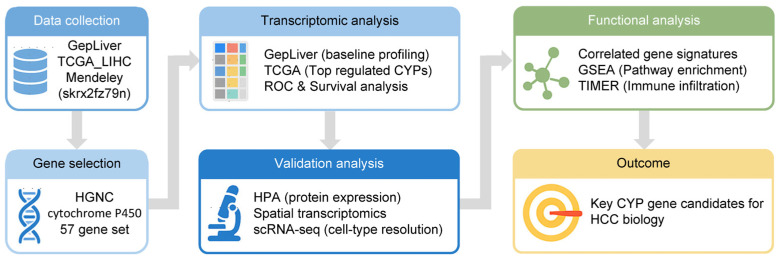
Workflow and transcriptomic profiling of CYP genes in HCC. Overall workflow of the study. CYP gene family members (n = 57) were defined based on HGNC annotation. Data were collected from multiple sources, including GepLiver and TCGA, followed by transcriptomic analysis to identify differentially expressed genes, validation using external resources (HPA, scRNA-seq, spatial transcriptomics), and functional characterization through pathway and immune analyses.

To systematically investigate the cytochrome P450 (CYP) gene family in hepatocellular carcinoma (HCC), we established an integrative multi-step analytic workflow encompassing large-scale data collection, rigorous gene set selection, transcriptomic profiling, external cohort validation, and downstream functional assessments (Figure 1). A total of 57 CYP genes were curated from the HGNC database and examined using the GepLiver cohort, providing a comprehensive survey of expression patterns across normal, adjacent non-tumor, and HCC tissues.

**Figure 2:**
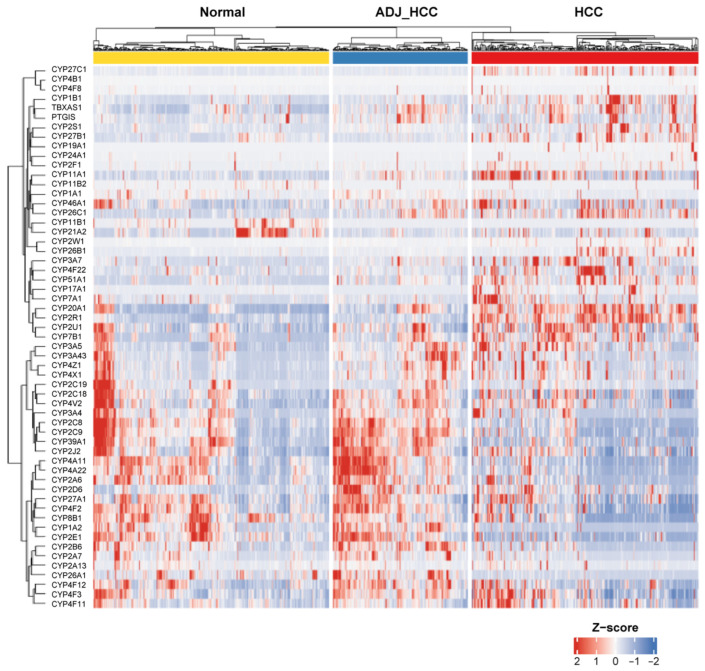
Transcriptomic profiling of CYP genes in GepLiver cohort. Heatmap of 57 CYP genes in the GepLiver cohort, stratified into Normal, adjacent non-tumor (ADJ_HCC), and HCC tissues. Gene expression values were normalized by z-score across samples. Distinct up- and down-regulation patterns were observed among CYP family members.

Unsupervised hierarchical clustering and heatmap visualization highlighted marked heterogeneity in CYP gene expression, with striking subgrouping according to tissue type and sample characteristics (Figure 2). Key hepatic CYP members, including *CYP3A4*, *CYP2E1*, and *CYP2C9*, were consistently down-regulated in HCC relative to control samples. By contrast, several genes, such as *CYP26B1*, *CYP1B1*, and *CYP7A1*, and especially *CYP2R1*, displayed robust up-regulation specific to tumor tissues. Deregulation thus manifested as gene-specific rather than family-wide shifts.

Complementing transcriptomic findings, we assessed genomic alterations using cBioPortal data from the TCGA-LIHC cohort (n = 348). Most CYP genes lacked recurrent mutations or structural variants, indicating that somatic genomic changes are rare across this family. Notably, copy-number amplifications were seen for *CYP11B1* and *CYP11B2* in a single subject, while *CYP2R1* exhibited a high frequency of mRNA overexpression events. Consistently, most CYP genes exhibited frequent mRNA low cases, mirroring transcriptomic down-regulation (Supplementary Figure S1).

**Figure 3:**
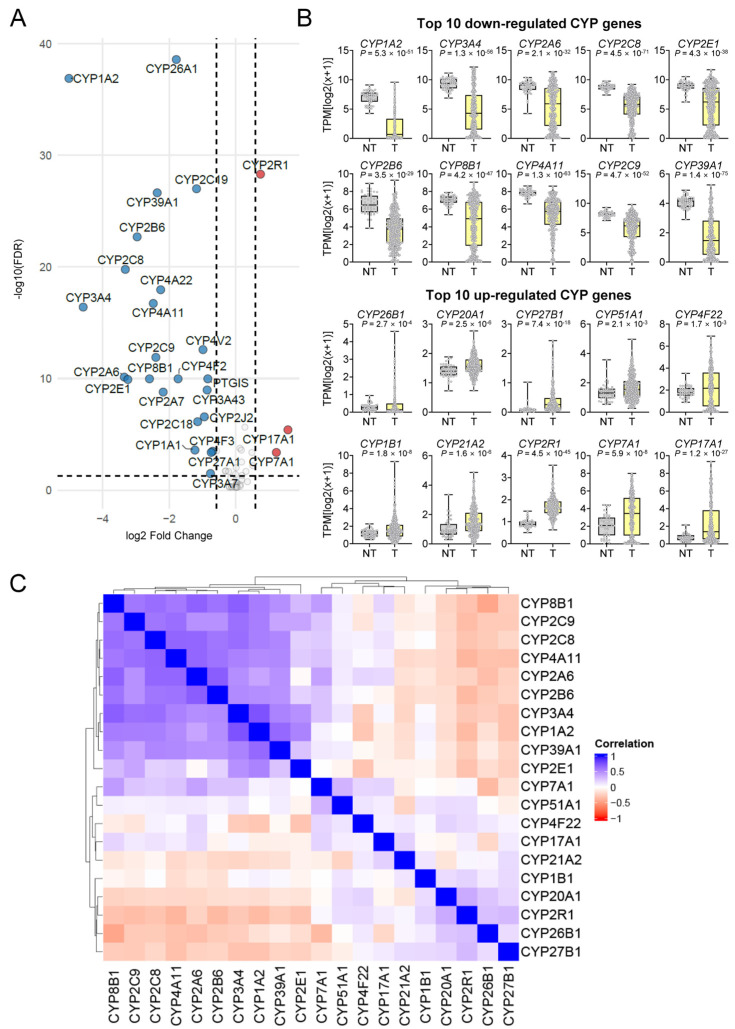
Identification of candidate CYP genes associated with HCC. (**A**) Volcano plot showing expressed CYP genes between HCC (T) and non-tumor (NT) tissues in the TCGA cohort, differentially. The dashed lines represent the significance thresholds, with vertical lines indicating|log2 fold change| > 0.585 (corresponding to a 1.5-fold change) and the horizontal line indicating FDR < 0.05. Significantly up- and down-regulated genes are highlighted. Colors represent upregulated (red) and downregulated (blue) genes. (**B**) Boxplots of the top 10 down-regulated and 10 up-regulated CYP genes, comparing expression between NT and T tissues. Expression values are represented as TPM [log2(X + 1)], and *p*-values were calculated using Welch’s *t*-test. Colors indicate NT (gray) and T (yellow). (**C**) Correlation heatmap of the top 20 differentially expressed CYP genes (10 up-regulated and 10 down-regulated) based on TCGA expression data. Pearson correlation coefficients are shown, with blue indicating positive and red indicating negative correlations.

To robustly validate differential expression, we performed volcano plot and boxplot analyses using TCGA data. Among these, *CYP2R1*, *CYP7A1*, and *CYP17A1* were markedly up-regulated, whereas *CYP1A2*, *CYP3A4*, and *CYP2C8* were strongly down-regulated (Figure 3A). Based on differential expression analysis in the TCGA-LIHC cohort, CYP genes were ranked according to statistical significance (*p* < 0.05) and magnitude of change, and the 20 most dysregulated genes with the largest absolute fold-change were selected for subsequent ROC screening. The top 10 up-regulated CYP genes (*CYP17A1*, *CYP7A1*, *CYP2R1*, *CYP21A2*, *CYP1B1*, *CYP4F22*, *CYP51A1*, *CYP27B1*, *CYP20A1*, and *CYP26B1*) and the top 10 down-regulated CYP genes (*CYP1A2*, *CYP3A4*, *CYP2A6*, *CYP2C8*, *CYP2E1*, *CYP2B6*, *CYP8B1*, *CYP4A11*, *CYP2C9*, and *CYP39A1*) were selected as candidates for further evaluation. Boxplot analyses confirmed robust and significant differences in expression between tumor and non-tumor tissues for each of these genes (Figure 3B).

Correlation analysis of the top 20 candidate CYP genes revealed subgroup-specific clustering patterns. Within the down-regulated CYP subset, the strongest positive correlations were observed for *CYP1A2*–*CYP3A4* (r = 0.75, *p* = 6.2 × 10^−77^), *CYP2A6*–*CYP2B6* (r = 0.70, *p* = 3.1 × 10^−63^), and *CYP3A4*–*CYP8B1* (r = 0.70, *p* = 3.5 × 10^−62^). Among the up-regulated CYPs, the most notable positive associations were *CYP20A1*–*CYP2R1* (r = 0.37, *p* = 9.2 × 10^−15^), *CYP51A1*–*CYP7A1* (r = 0.33, *p* = 4.5 × 10^−12^), and *CYP27B1*–*CYP2R1* (r = 0.30, *p* = 4.0 × 10^−10^). By contrast, cross-group correlations were generally weak or negative, with the strongest inverse relationships identified between *CYP8B1* (down) and *CYP26B1* (up) (r = –0.46, *p* = 2.4 × 10^−23^), *CYP4A11* (down) and *CYP2R1* (up) (r = –0.38, *p* = 5.8 × 10^−16^), and *CYP2B6* (down) and *CYP2R1* (up) (r = –0.36, *p* = 2.2 × 10^−14^). These findings highlight tightly coordinated expression within up- and down-regulated CYP subsets, while underscoring divergent regulatory mechanisms between the two groups (Figure 3C, Supplementary Table S1).

Collectively, these analyses underscore the context-dependent and gene-specific deregulation of CYP family members in HCC, highlighting *CYP2R1* and a select group of genes as the most prominent candidates for further functional and clinical investigation.

### 3.2. Diagnostic Performance of Candidate CYP Genes in HCC

**Figure 4:**
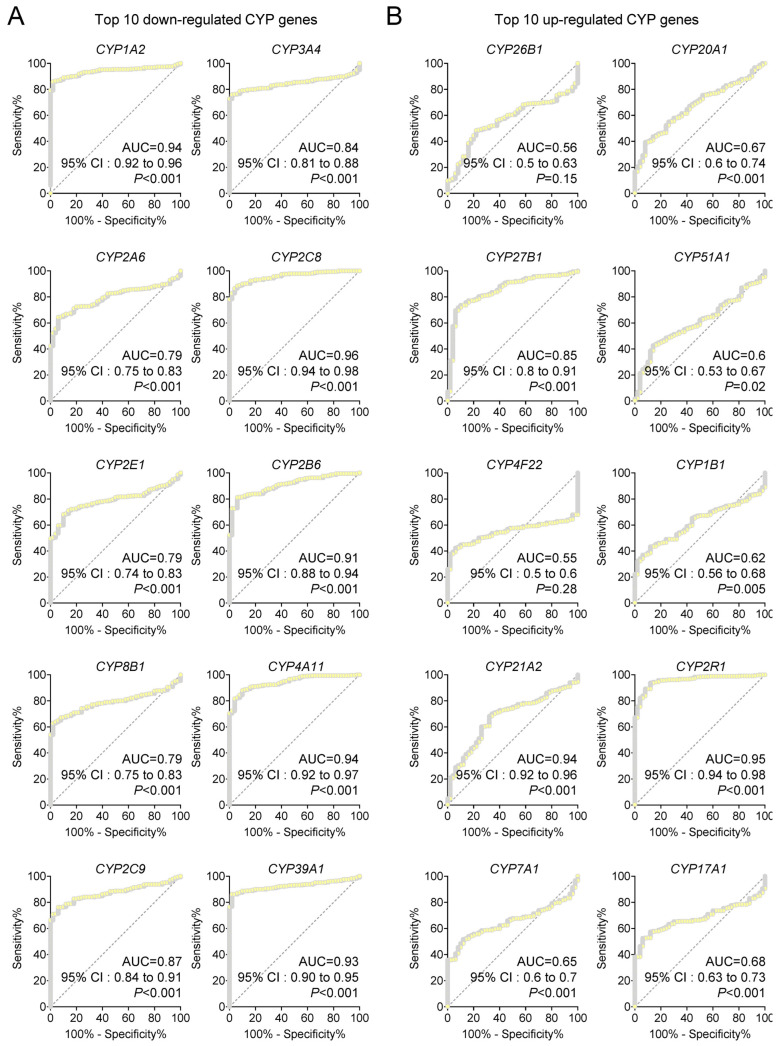
Diagnostic performance of top 20 differentially expressed CYP genes in TCGA cohort. Receiver operating characteristic (ROC) analyses were conducted for the top 10 down-regulated (**A**) and top 10 up-regulated (**B**) CYP genes, comparing HCC tissues (T, n = 371) with non-tumor controls (NT, n = 50). Area under the curve (AUC) values, 95% confidence intervals (CI), and significance levels were computed to evaluate diagnostic accuracy. The dashed line represents the reference line for random classification (AUC = 0.5).

To determine the diagnostic value of the most dysregulated CYP family members, we conducted receiver-operating characteristic (ROC) curve analyses for the top 20 differentially expressed CYP genes using the TCGA-LIHC cohort, consisting of 371 tumor and 50 non-tumor samples (Figure 4).

Within the down-regulated subset, five genes exhibited excellent discriminatory power with AUC values greater than 0.90, including *CYP1A2* (AUC = 0.94, 95% CI = 0.92–0.96, *p* < 0.001), *CYP2C8* (AUC = 0.96, 95% CI = 0.94–0.98, *p* < 0.001), *CYP2B6* (AUC = 0.91, 95% CI = 0.88–0.94, *p* < 0.001), *CYP4A11* (AUC = 0.94, 95% CI = 0.92–0.97, *p* < 0.001), and *CYP39A1* (AUC = 0.93, 95% CI = 0.90–0.95, *p* < 0.001). *CYP2C8* demonstrated the strongest performance, with an AUC of 0.96 and a narrow 95% confidence interval (0.94–0.98), highlighting its powerful ability to differentiate HCC from non-tumor tissues.

Among the up-regulated genes, *CYP21A2* (AUC = 0.94, 95% CI = 0.92–0.96, *p* < 0.001) and *CYP2R1* (AUC = 0.95, 95% CI = 0.94–0.98, *p* < 0.001) also showed high diagnostic accuracy. In particular, *CYP2R1* emerged as the top up-regulated diagnostic marker, matching down-regulated *CYP2C8* in performance. Other CYPs demonstrated moderate to good diagnostic capacity, with AUCs and statistical metrics summarized in Supplementary Table S2 for all 20 candidate genes.

These findings firmly establish *CYP2C8* (down-regulated) and *CYP2R1* (up-regulated) as the most robust diagnostic biomarkers in the CYP family for HCC, offering high accuracy, reproducibility, and clinical applicability across independent patient datasets.

### 3.3. Prognostic Significance of CYP Gene Expression in HCC

**Figure 5:**
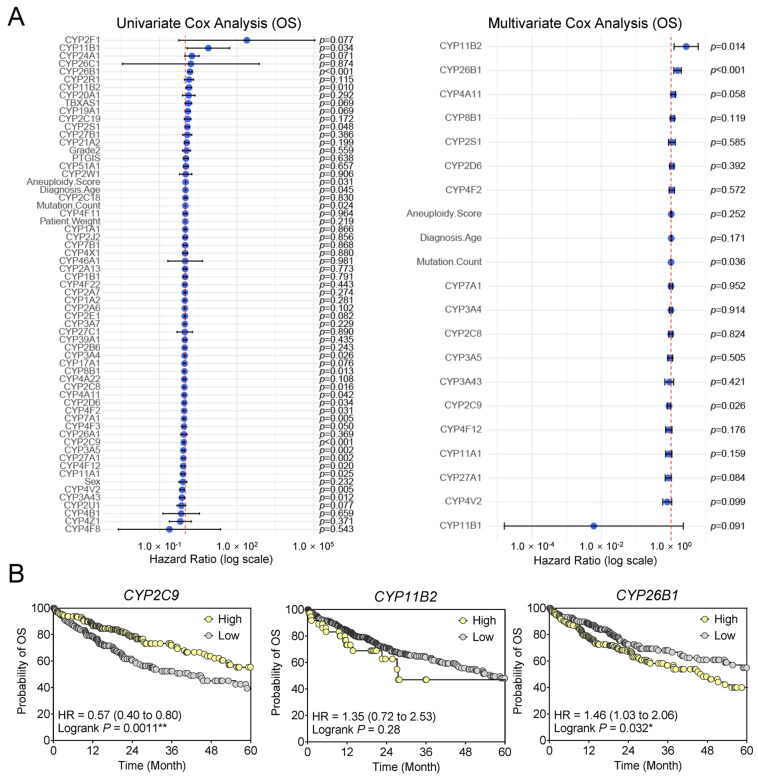
Prognostic relevance of CYP genes in HCC. (**A**) Forest plots from univariate and multivariate Cox regression analyses for overall survival (OS). Univariate analysis included six clinical variables (Diagnosis Age, Sex, Patient Weight, Aneuploidy Score, Mutation Count, and Histologic Grade [G1–2 vs. G3–4]) and 57 CYP genes. Nineteen variables showed significance at *p* < 0.05 in the univariate model, including three clinical factors (Diagnosis Age, Aneuploidy Score, Mutation Count) and 16 CYP genes. These significant variables were subsequently included in a multivariate Cox regression model, in which four factors (Mutation Count, *CYP2C9*, *CYP11B2*, and *CYP26B1*) remained significant (*p* < 0.05). The red dashed line represents the reference line (hazard ratio = 1). Blue dots indicate hazard ratios, and horizontal lines represent 95% confidence intervals. (**B**) Kaplan–Meier survival analyses for the three CYP genes identified in the multivariate model. * *p* < 0.05; ** *p* < 0.01.

To determine the prognostic significance of CYP family members in HCC, Cox proportional hazards regression analyses were performed on TCGA cohort data. Univariate Cox analysis included six clinical variables (Diagnosis Age, Sex, Patient Weight, Aneuploidy Score, Mutation Count, and Histologic Grade [G1–2 vs. G3–4]) and 57 CYP genes (Figure 5A, Supplementary Table S3). A total of 19 factors demonstrated prognostic significance (*p* < 0.05). Among the clinical variables, only Diagnosis Age, Aneuploidy Score, and Mutation Count were identified, although their hazard ratios (HRs) were close to 1, suggesting limited prognostic impact.

In the univariate analysis of CYP genes, *CYP11B1*, *CYP11B2*, and *CYP2S1* exhibited HRs greater than 1, consistent with poor overall survival (OS). In contrast, multiple CYPs correlated with favorable prognosis (HR < 1), including *CYP3A4*, *CYP2C8*, *CYP8B1*, *CYP4A11*, *CYP4F2*, *CYP4V2*, *CYP3A43*, *CYP27A1*, *CYP4F12*, *CYP3A5*, *CYP2D6*, *CYP11A1*, and *CYP7A1*.

The multivariate Cox model, incorporating the 19 significant univariate factors, identified four independent predictors for OS: Mutation Count (HR = 1.00, 95% CI: 1.00–1.00, *p* = 0.036), *CYP2C9* (HR = 0.88, 95% CI: 0.78–0.98, *p* = 0.026), *CYP26B1* (HR = 1.54, 95% CI: 1.20–1.98, *p* = 0.001), and *CYP11B2* (HR = 2.73, 95% CI: 1.22–6.07, *p* = 0.014). These findings highlight that both clinical genomic burden (Mutation Count) and specific CYP family members contribute to survival outcomes in HCC (Supplementary Table S4). The final multivariable model achieved a concordance index of 0.669, indicating moderate concordance between predicted and observed survival.

Kaplan–Meier survival analyses further illustrated the clinical relevance of these CYPs. Patients with high *CYP2C9* expression exhibited significantly improved OS compared with the low-expression group (HR = 0.57, 95% CI: 0.40–0.80, log-rank *p* = 0.0011). In contrast, high *CYP26B1* expression was associated with significantly poorer OS (HR = 1.46, 95% CI: 1.03–2.06, log-rank *p* = 0.032). For *CYP11B2*, dichotomized analysis (zero vs. non-zero expression) suggested a modest prognostic trend (HR = 1.35, 95% CI: 0.72–2.53, log-rank *p* = 0.28), though expression was nearly absent in the majority of cases (n = 330) (Figure 5B).

Collectively, these findings establish *CYP2C9* (favorable) and *CYP26B1* (unfavorable) as independent prognostic biomarkers for HCC, whereas the prognostic significance of *CYP11B2* is limited by sparse hepatic expression and skewed distribution.

### 3.4. Comprehensive Validation of CYP2R1 Overexpression and Diagnostic Efficacy in HCC

To assess protein expression of candidate CYP biomarkers, we performed immunohistochemical analysis using the Human Protein Atlas and tissue microarrays for five genes: *CYP2C8*, *CYP2C9*, *CYP2R1*, *CYP26B1*, and *CYP11B2* (Supplementary Figure S2). *CYP2C8* and *CYP2C9* showed marked reduction in HCC, matching their previously reported diagnostic and prognostic significance in liver cancer. However, as both are already well-established markers in hepatocellular carcinoma [10,16], they were not selected for further novelty-based validation. CYP11B2 and CYP26B1 did not show a significant difference between tumor and non-tumor tissue at the protein level, so they were excluded as practical tissue biomarkers. In contrast, CYP2R1 demonstrated robust and significant overexpression in HCC at both the transcript and protein levels (*p* < 0.001), consistently validated across platforms, and was prioritized as the novel candidate for detailed analysis.

**Figure 6:**
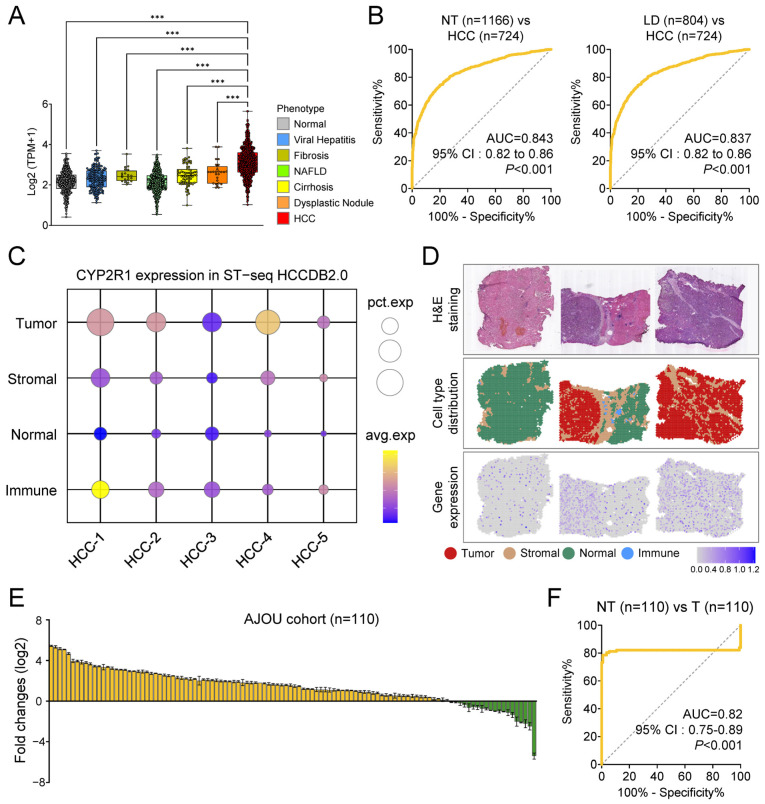
Validation of *CYP2R1* overexpression and diagnostic performance in HCC. (**A**) *CYP2R1* expression analysis in the GepLiver cohort. *** *p* < 0.001. (**B**) ROC curves demonstrating the diagnostic performance of *CYP2R1* for distinguishing HCC from non-tumor tissue, liver disease (LD), and HCC in GepLiver cohort. (**C**) Spatial expression of *CYP2R1* across tumor and surrounding regions in HCC samples. The plot shows the percentage of *CYP2R1* expression (pct.exp) across spatial regions. The color scale represents the average expression level of *CYP2R1*. (**D**) TMA images representing *CYP2R1* expression in HCC tissues. The scale bar represents the expression level, with higher expression shown in yellow and lower expression in purple. (**E**) Fold change in *CYP2R1* expression (log2) between non-tumor (NT) and tumor (T) tissues in 110 HCC patients from the Ajou hospital cohort. Statistical analysis was performed using the paired *t*-test. Colors indicate downregulation (green) and upregulation (yellow). (**F**) ROC analysis of *CYP2R1* expression between NT and T in 110 HCC patients from the Ajou hospital cohort.

Given the unique and consistent upregulation of *CYP2R1* in HCC, we further validated its clinical utility across diverse patient cohorts and biological contexts. In the GepLiver cohort, *CYP2R1* expression was markedly elevated in HCC compared to all other groups, including normal liver, viral hepatitis, NAFLD, fibrosis, cirrhosis, and dysplastic nodules (Figure 6A). *CYP2R1* demonstrated strong diagnostic accuracy, with AUC = 0.843 (95% CI: 0.82–0.86, *p* < 0.001) for distinguishing HCC from non-tumor tissues, and similar performance after excluding normal controls (AUC = 0.837, 95% CI = 0.82–0.86, *p* < 0.001) (Figure 6B).

Spatial transcriptomics using the HCCDB2.0 resource localized *CYP2R1* overexpression specifically to malignant hepatocyte (tumor) regions, with minimal signal in the surrounding stromal, immune, or normal compartments (Figure 6C,D). Furthermore, matched tumor and adjacent tissue samples from an independent Ajou University Hospital cohort (n = 110) showed significant overexpression of *CYP2R1* in HCC by qRT-PCR (Figure 6E), with ROC analysis yielding an AUC of 0.82 (95% CI = 0.75–0.89, *p* < 0.001), confirming the biomarker’s reproducibility across populations (Figure 6F). To assess potential confounding by tumor burden, *CYP2R1* expression was evaluated across BCLC and pathological UICC stages in the Ajou validation cohort and showed no significant stage-dependent differences (Supplementary Figure S3A). Cohort characteristics are summarized in Supplementary Table S6, and the paired tumor–adjacent design enabled intra-individual comparison under a standardized institutional protocol, minimizing patient-level confounding. In an exploratory subset with paired primary and recurrent tumors (n = 6), *CYP2R1* expression showed heterogeneous changes at recurrence (Supplementary Figure S3B). In addition, tumors with vascular invasion exhibited higher *CYP2R1* expression than those without vascular invasion (Supplementary Figure S3C). An exploratory ELISA analysis of plasma samples showed modest discrimination between hepatocellular carcinoma and non-tumor liver disease controls (AUC = 0.71), suggesting that circulating *CYP2R1* may be detectable but requires further validation for clinical application (Supplementary Figure S3D). Taken together, these findings provide cross-platform, multi-cohort evidence that *CYP2R1* is consistently and specifically upregulated in HCC, supporting its potential utility as a novel diagnostic biomarker across independent cohorts.

### 3.5. Functional Characterization of CYP2R1-Associated Pathways in HCC

**Figure 7:**
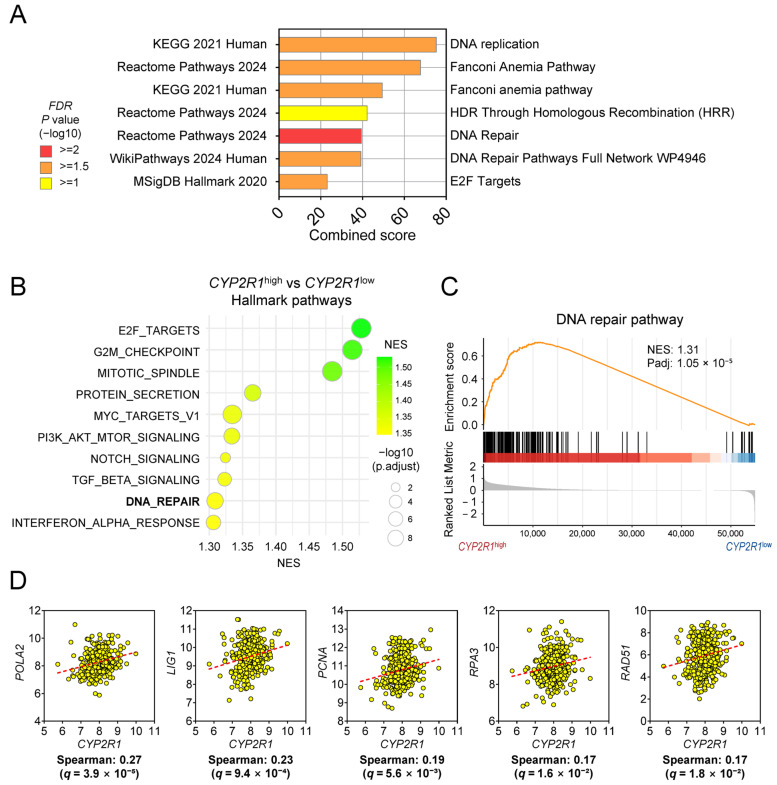
Functional characterization of *CYP2R1*-associated pathways in HCC. (**A**) Bar plot summarizing enriched pathways that remained significant after multiple-testing correction (FDR-adjusted *p* < 0.05) across four databases (KEGG 2021 Human, Reactome Pathways 2024, MSigDB Hallmark 2020, and WikiPathways 2024 Human). Bars represent the combined enrichment score for each pathway, and colors indicate the FDR-adjusted *p* value on a –log10 scale (yellow ≥ 1, orange ≥ 1.5, red ≥ 2). (**B**) Top 10 Hallmark pathways identified by GSEA comparing *CYP2R1*-high (Q1) vs. *CYP2R1*-low (Q4) TCGA HCC samples. (**C**) GSEA enrichment plot of the DNA repair program. The orange line represents the enrichment score. (**D**) Correlation plots of *CYP2R1* expression with representative DNA repair genes (*POLA2*, *LIG1*, *PCNA*, *RPA3*, and *RAD51*). The dashed lines represent fitted regression lines.

To elucidate the biological programs linked to *CYP2R1* expression, we analyzed co-expression networks using cBioPortal. A total of 594 genes significantly correlated with *CYP2R1* (r ≥ |0.2|, q < 0.05) were subjected to enrichment analysis with EnrichR. Across the four enrichment databases, several pathways remained statistically significant after multiple-testing correction (FDR-adjusted *p* < 0.05). Among these, DNA replication was among the top-enriched biological processes associated with the *CYP2R1* co-expression network. In particular, pathways related to DNA replication, base excision repair, homologous recombination, and general DNA repair processes showed strong enrichment signals. In addition, the E2F Targets signature from the MSigDB Hallmark collection was also identified as a significantly enriched pathway. These findings collectively indicate that *CYP2R1*-associated transcriptional programs are closely linked to cell-cycle regulation and DNA repair–related biological processes (Figure 7A, Supplementary Figure S4).

To validate these enrichment findings, gene set enrichment analysis (GSEA) was performed on TCGA-LIHC data stratified by *CYP2R1* expression quartiles. Differential expression analysis between *CYP2R1*-high (Q1) and *CYP2R1*-low (Q4) tumors was used to generate a ranked gene list for Hallmark gene set enrichment. Among the top 10 enriched pathways, DNA Repair was again identified as significantly upregulated in the *CYP2R1*-high group (Figure 7B). The enrichment curve confirmed robust activation of DNA repair programs in the *CYP2R1*-high group (NES = 1.31, adjusted *p* = 1.05 × 10^−5^) (Figure 7C).

Correlation analysis supported these results, showing that *CYP2R1* expression positively correlated with key DNA replication and repair factors: *POLA2* (r = 0.27, q = 3.9 × 10^−5^), *LIG1* (r = 0.23, q = 9.4 × 10^−4^), *PCNA* (r = 0.19, q = 5.6 × 10^−3^), *RPA3* (r = 0.17, q = 1.6 × 10^−2^), and *RAD51* (r = 0.17, q = 1.8 × 10^−2^) (Figure 7D). *POLA2* and *LIG1* are essential for the initiation of DNA replication and repair [17,18], while *PCNA* and *RPA3* play central roles in replication fork stability and repair fidelity [19,20]. *RAD51* is a key mediator of homologous recombination repair [21]. The consistent co-expression of *CYP2R1* with these core DNA repair genes suggests that high *CYP2R1* expression is closely linked to enhanced activation of DNA replication and repair machinery in HCC, potentially contributing to the maintenance of genomic stability in tumor cells.

### 3.6. CYP2R1 Expression Is Closely Associated with MDSC Infiltration and Marker Gene Signature in HCC

**Figure 8:**
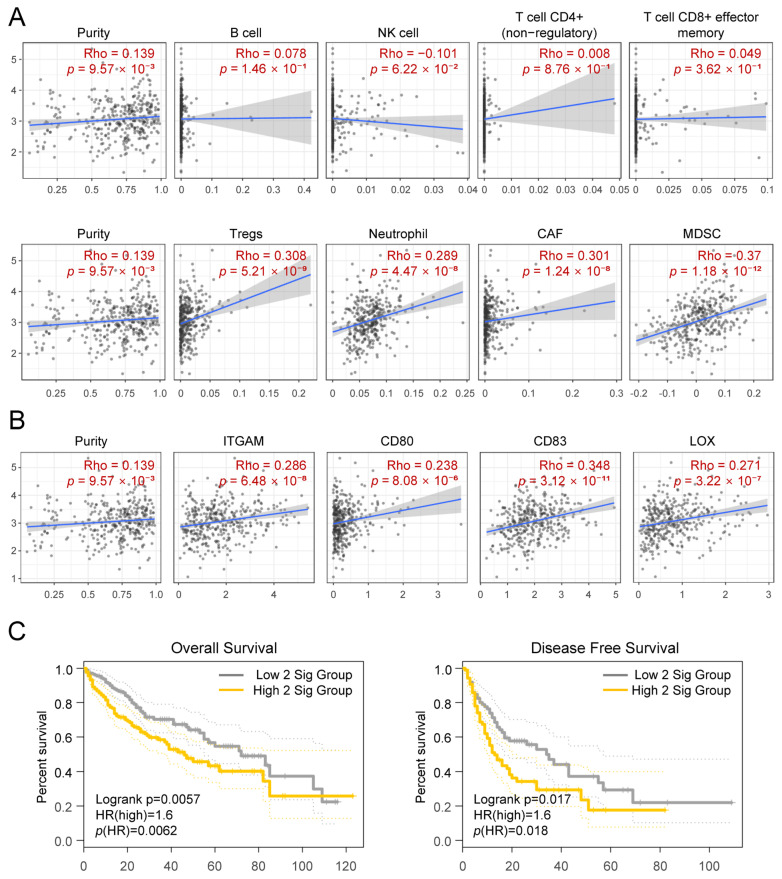
Association of *CYP2R1* expression with immune infiltration and clinical outcome in HCC. (**A**) Correlation analysis between *CYP2R1* expression and tumor-infiltrating immune cell populations using TIMER2.0. Blue lines represent fitted regression lines, and shaded areas indicate confidence intervals. (**B**) Correlation between *CYP2R1* and representative MDSC marker genes based on the TCGA-LIHC dataset. Blue lines represent fitted regression lines, and shaded areas indicate confidence intervals. (**C**) Kaplan–Meier survival analysis using the GEPIA platform showing overall survival (OS, **left**) and disease-free survival (DFS, **right**) according to combined *CYP2R1* and *CD83* expression levels. Yellow and gray lines represent different expression groups (high vs. low).

To investigate the immunological context of *CYP2R1* expression in HCC, we utilized the TIMER2.0 platform to perform correlation analyses between *CYP2R1* and various tumor-infiltrating immune cell populations (Figure 8A). Among lymphoid and myeloid subtypes, the strongest positive association was observed between *CYP2R1* expression and myeloid-derived suppressor cell (MDSC) infiltration (r = 0.37, *p* = 1.18 × 10^−12^). Correlations with other major immune cell populations, including B cells, NK cells, CD4+ and CD8+ T cells, were weak or non-significant.

To further dissect the relationship between *CYP2R1* and the immunosuppressive microenvironment, we analyzed the expression of established MDSC marker genes. Among eleven evaluated markers, several exhibited statistically significant positive correlations with *CYP2R1*. Notably, *CD83* showed the highest correlation (r = 0.348, *p* = 3.12 × 10^−11^), followed by *LOX* (r = 0.271, *p* = 3.22 × 10^−7^), *ITGAM* (r = 0.286, *p* = 6.48 × 10^−8^) (Figure 8B). Full correlation results for all eleven markers are provided in Supplementary Table S5.

To further evaluate the prognostic implications of the *CYP2R1*–*CD83* axis, we performed Kaplan–Meier survival analyses using the GEPIA platform. While *CYP2R1* or *CD83* expression alone did not significantly affect overall or disease-free survival, their combined high expression was significantly associated with poorer patient outcomes. Specifically, in the OS analysis, the *CYP2R1*–*CD83* high co-expression group exhibited significantly shorter survival compared with other groups (log-rank *p* = 0.0057; HR (high) = 1.6; *p* (HR) = 0.0062). Similarly, disease-free survival (DFS) analysis revealed a consistent trend, with the high co-expression group showing reduced recurrence-free survival (log-rank *p* = 0.017; HR (high) = 1.6; *p* (HR) = 0.018) (Figure 8C).

These findings suggest that high *CYP2R1* expression in HCC is linked to increased infiltration and molecular signatures of MDSCs, emphasizing a potential immunosuppressive niche associated with *CYP2R1* upregulation. The observed *CYP2R1*–*CD83* co-expression-dependent reduction in both overall and disease-free survival further supports the clinical relevance of this metabolic–immune interaction axis in HCC.

Given the established role of MDSCs in tumor immune evasion and resistance to immunotherapy, this *CYP2R1*–MDSC axis highlights a biologically and clinically meaningful connection warranting further mechanistic and translational investigation.

## 4. Discussion

Cytochrome P450 enzymes are central regulators of hepatic metabolism, yet their collective contribution to hepatocarcinogenesis remains incompletely understood. By integrating multi-cohort transcriptomic datasets, survival analyses, and functional inference, this study delineates global dysregulation of CYP family genes in HCC and identifies distinct diagnostic and prognostic signatures. Among these, *CYP2R1* emerged as a novel tumor-associated CYP with both diagnostic and potential mechanistic relevance, while *CYP2C9* and *CYP26B1* demonstrated opposing prognostic implications. These findings suggest that CYP reprogramming in HCC is gene-specific and reflects the interplay between metabolic, genomic, and signaling alterations during liver tumor development.

Among the CYP genes analyzed, *CYP2R1* showed the strongest diagnostic potential. It was the most significantly up-regulated gene in HCC and demonstrated the highest diagnostic accuracy (AUC = 0.95). *CYP2R1* encodes a microsomal vitamin D 25-hydroxylase that converts vitamin D to 25-hydroxyvitamin D, the major circulating biomarker of vitamin D status [22]. Although its physiological hepatic role is well established, its involvement in liver tumorigenesis has not previously been characterized. The upregulation of *CYP2R1* may extend beyond vitamin D metabolism. The vitamin D–VDR signaling axis has been reported to exert antiproliferative, anti-inflammatory, and DNA-protective effects in the liver [23], and *CYP2R1* functions as a key enzyme in this pathway. Dysregulated *CYP2R1* expression may therefore reflect metabolic adaptation to oncogenic stress, linking hepatic metabolism to genomic maintenance and immune regulation in HCC.

*CYP2C9* emerged as a reliable prognostic marker. In both univariate and multivariate Cox regression analyses, higher *CYP2C9* expression was consistently associated with favorable overall survival. Previous studies have reported reduced *CYP2C9* expression in HCC tissues compared with adjacent non-tumor liver, suggesting that its loss of hepatic metabolic capacity may contribute to disease progression and poorer clinical outcomes [24]. Our findings reinforce *CYP2C9* as a prognostically relevant CYP gene in HCC.

In contrast, *CYP26B1* expression correlated with poorer survival. *CYP26B1* catalyzes the degradation of retinoic acid, a signaling molecule essential for cellular differentiation and growth. Dysregulation of retinoic acid metabolism has been implicated in hepatocarcinogenesis [25,26], and increased *CYP26B1* expression may promote tumor progression by suppressing retinoid signaling. Although studies examining *CYP26B1* in HCC remain limited, our results suggest that it may represent a candidate prognostic biomarker. It should also be noted, however, that the Cox regression analyses in this study were designed as exploratory modeling within the TCGA-LIHC cohort to identify candidate prognostic CYP genes rather than to construct a fully optimized clinical prediction model.

Other CYP genes provided additional context. *CYP2C8* showed excellent diagnostic performance but was markedly downregulated in HCC, limiting its translational potential as a practical biomarker. Conversely, the apparent prognostic signal for *CYP11B2* is likely artifactual. *CYP11B2* encodes aldosterone synthase and is primarily expressed in the adrenal cortex [27]. The near-absent expression in most HCC samples suggests that the observed survival association likely reflects technical variability rather than biological relevance.

Importantly, *CYP2R1* upregulation did not appear to depend on tumor stage. In the validation cohort, *CYP2R1* expression did not significantly differ across BCLC or modified UICC stages, indicating that its induction represents a stage-independent molecular feature of HCC rather than a secondary consequence of tumor progression. In a small exploratory subset with paired primary and recurrent tumors, *CYP2R1* expression showed heterogeneous changes at recurrence, suggesting that its regulation during tumor evolution may vary between patients.

Our integrative analysis also revealed an association between *CYP2R1* expression and transcriptional programs related to DNA replication and repair. *CYP2R1* expression correlated positively with key replication and repair genes, including *POLA2*, *LIG1*, and *PCNA*. *POLA2* participates in replication initiation through DNA polymerase α complexes [28]. *LIG1* is essential for Okazaki fragments ligation and base excision repair [29], and PCNA functions as a central processivity factor coordinating DNA replication and repair fidelity [30]. These observations suggest that CYP2R1-high tumors may exhibit enhanced transcriptional activity in DNA maintenance pathways.

Because *CYP2R1* participates in microsomal oxidation reactions capable of generating reactive oxygen species (ROS), it is possible that increased *CYP2R1* expression may be associated with compensatory activation of DNA repair pathways. However, the present transcriptomic analyses reveal correlations rather than causal relationships, and experimental studies will be required to determine whether *CYP2R1* directly influences DNA repair processes. Vitamin D–VDR signaling has also been reported to regulate DNA repair genes such as *RAD51* and *LIG1* via p53-dependent mechanisms [31], raising the possibility that CYP2R1-associated vitamin D metabolism may influence genomic maintenance pathways. These observations should therefore be considered hypothesis-generating and require functional validation.

In addition to genomic maintenance, our analyses suggest a potential immunological dimension to *CYP2R1* activity. TIMER-based immune infiltration analysis revealed a positive correlation between *CYP2R1* expression and myeloid-derived suppressor cell (MDSC) abundance, along with co-expression of MDSC-associated genes, including *CD83*, *LOX*, *ITGAM*, and CD80. Because MDSCs suppress antitumor immunity and contribute to immune checkpoint resistance [32], these findings raise the possibility that CYP2R1-associated metabolic changes may contribute to an immunosuppressive tumor microenvironment. However, these immune infiltration estimates were derived from transcriptomic deconvolution methods and should therefore be interpreted cautiously. Computational approaches such as TIMER2.0 infer immune cell abundance indirectly from bulk RNA-seq data and cannot capture spatial immune architecture. Future studies using spatial transcriptomics or immunohistochemical validation will be required to confirm the relationship between *CYP2R1* expression and MDSC infiltration.

Several limitations should be considered when interpreting the present findings. Public transcriptomic datasets such as TCGA-LIHC and GepLiver lack detailed information on certain potential confounding variables, including medication exposure, alcohol consumption, metabolic comorbidities, and tissue procurement conditions. Because CYP enzymes are inducible by environmental and pharmacological factors, complete matching across disease groups was not feasible. Nevertheless, our findings were consistently reproduced across independent cohorts and further supported by a paired validation cohort from Ajou University Hospital, which minimized inter-individual variability. Future studies incorporating proteomic or antibody-based validation will further clarify the biological role of *CYP2R1* in HCC.

In summary, this study identifies *CYP2R1* as a promising metabolic and immunogenomic biomarker candidate and highlights *CYP2C9* and *CYP26B1* as prognostically relevant CYP genes in HCC. These findings provide insight into the heterogeneous roles of CYP family members in liver cancer and suggest that metabolic enzymes may contribute to tumor-associated genomic and immune adaptations. *CYP2R1* may represent a complementary tissue-based biomarker reflecting metabolic alterations in HCC. However, direct head-to-head comparisons with established serum biomarkers such as AFP and PIVKA-II were not available in the current retrospective cohorts, and future studies integrating tissue and circulating biomarkers will be required to determine their combined clinical utility.

## Data Availability

The transcriptomic and clinical datasets analyzed in this study are publicly available from The Cancer Genome Atlas Liver Hepatocellular Carcinoma (TCGA-LIHC; https://portal.gdc.cancer.gov/ (accessed on 19 February 2026)), GepLiver (http://www.gepliver.org (accessed on 19 February 2026)), and HCCDB2.0 databases (http://lifeome.net/database/hccdb/home.html (accessed on 19 February 2026)). Immunohistochemistry data were obtained from the Human Protein Atlas (HPA; https://www.proteinatlas.org/ (accessed on 19 February 2026)). Clinical samples and associated data from Ajou University Hospital were provided by the Ajou Human Source Bank and are not publicly available due to ethical and legal restrictions but are available from the corresponding author upon reasonable request.

## Supplementary Materials

The following supporting information can be downloaded at: https://www.mdpi.com/article/10.3390/medsci14020178/s1. Figure S1: Genomic and transcriptomic alterations of CYP genes using cBioPortal; Figure S2: Comparative immunohistochemical analysis of CYP protein expression in normal liver and hepatocellular carcinoma (HCC) tissues; Figure S3: Clinical and plasma correlates of *CYP2R1* expression in HCC; Figure S4: Pathway enrichment profiles associated with the *CYP2R1* co-expression network across four databases; Table S1: Pearson correlation coefficients and corresponding *p*-values for the top 20 differentially expressed CYP genes (10 up-regulated and 10 down-regulated) in the TCGA HCC cohort; Table S2: Receiver operating characteristic (ROC) analyses of the top 20 differentially expressed CYP genes in TCGA cohort; Table S3: Univariate Cox proportional hazards analysis of clinical variables and CYP gene expression in TCGA HCC cohort; Table S4: Multivariate Cox proportional hazards analysis of significant clinical variables and CYP gene expression in TCGA HCC cohort; Table S5:Correlation of *CYP2R1* expression with eleven canonical MDSC marker genes in the TCGA-LIHC cohort; Table S6: Baseline clinicopathological features of the tissue cohort.
