# Supplementary material for: Comprehensive Multi-Omics Characterization of CYP2R1 as a Diagnostic and Functional Biomarker in Hepatocellular Carcinoma
Source: Med Sci (Basel). 2026 Apr 1;14(2):178. doi: 10.3390/medsci14020178 (PMC13108089; doi:10.3390/medsci14020178)
Supplement: Supplementary file 1 [file medsci-14-00178-s001.zip › medsci-4188546-supplementary.pdf]

Supplementary information

Supplementary figures

Fig. S1

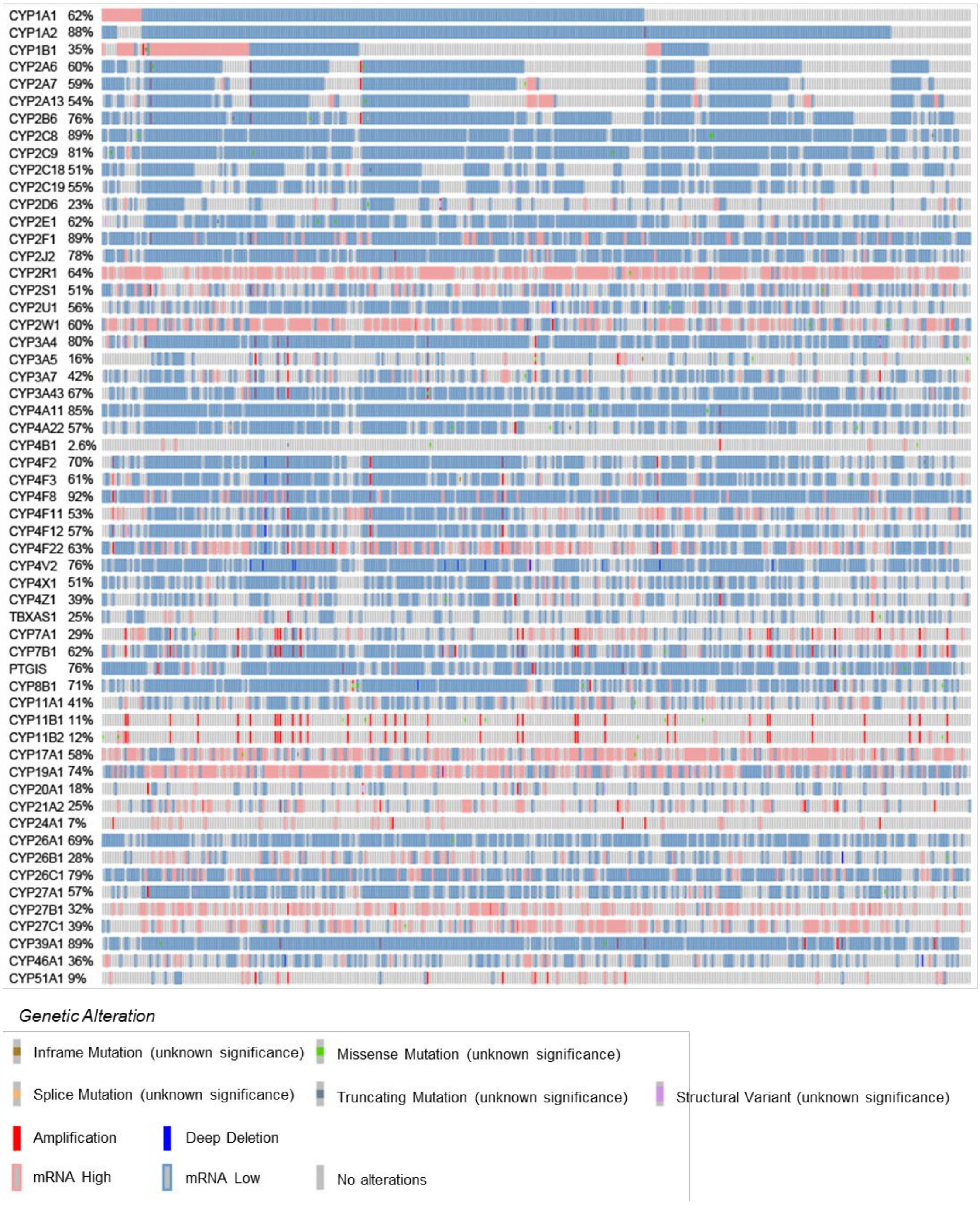

Supplementary Figure S1. Genomic and transcriptomic alterations of CYP genes using

cBioPortal.

OncoPrint plot of 57 CYP genes from the TCGA Liver Hepatocellular Carcinoma (PanCancer Atlas) study (n=348 complete samples). Genomic profiles included mutations, structural variants, and copy-number alterations. Most CYP genes showed few or no genomic alterations. Amplifications were detected for *CYP11B1* and *CYP11B2* in the same patient. mRNA expression z-scores (relative to normal samples; RNA Seq V2 RSEM, cutoff ±2.0) indicated that *CYP2R1* was frequently high, whereas the majority of CYP genes displayed a predominance of mRNA low cases, consistent with transcriptional down-regulation observed in the GepLiver dataset.

Fig. S2

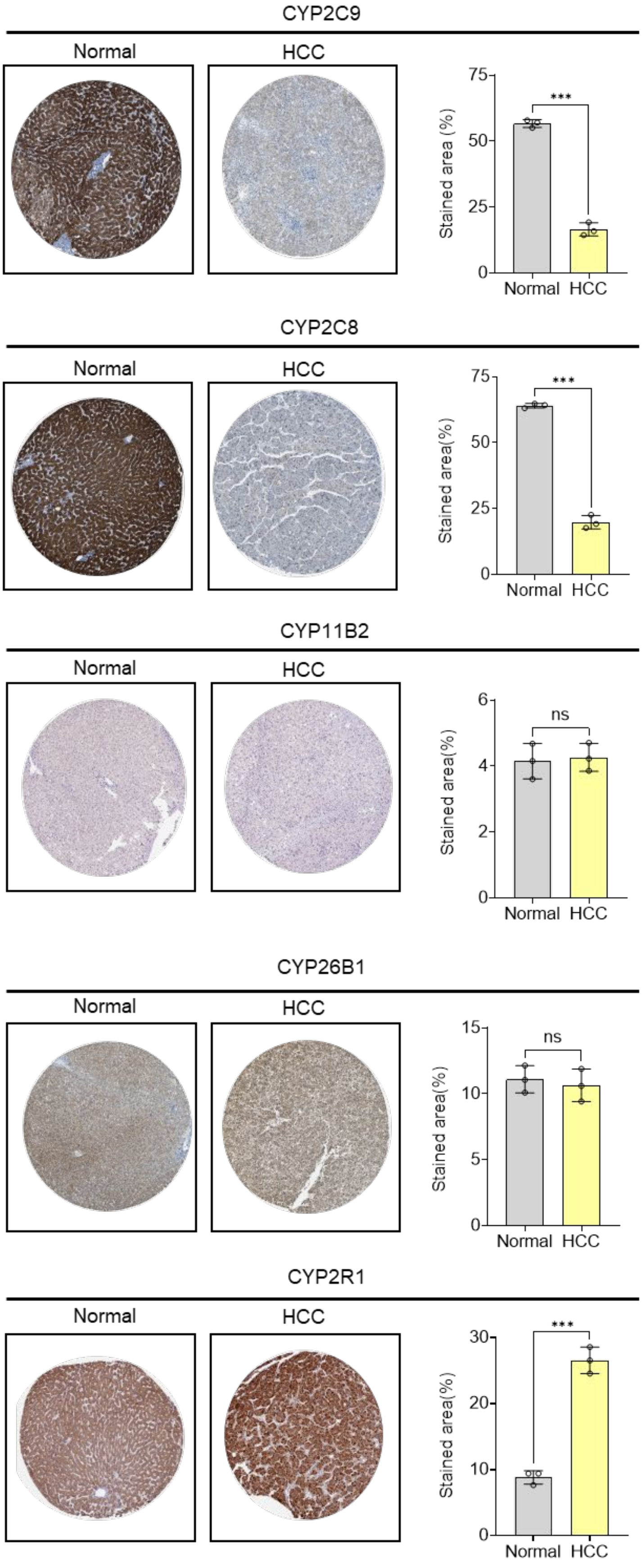
CYP2C9
Normal
HCC
Stained area (%)
75
50
25
0
***
Normal
HCC
CYP2C8
Normal
HCC
Stained area(%)
75
50
25
0
***
Normal
HCC
CYP11B2
Normal
HCC
Stained area(%)
6
4
2
0
ns
Normal
HCC
CYP26B1
Normal
HCC
Stained area(%)
15
10
5
0
ns
Normal
HCC
CYP2R1
Normal
HCC
Stained area(%)
30
20
10
0
***
Normal
HCC

Supplementary Figure S2. Comparative immunohistochemical analysis of CYP protein expression in normal liver and hepatocellular carcinoma (HCC) tissues.

Representative tissue microarray images and quantification of stained area (%) for five candidate CYP genes (*CYP2C9, CYP2C8*, *CYP11B2, CYP26B1*, and *CYP2R1*) in normal and HCC specimens.

Fig. S3

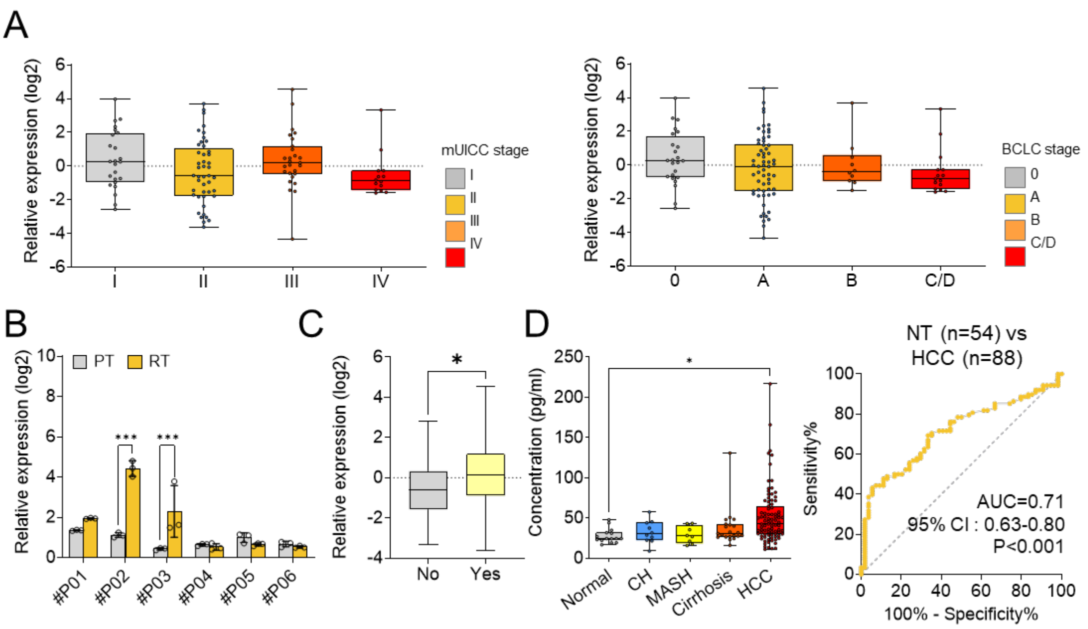

Figure S3. Clinical and plasma correlates of CYP2R1 expression in HCC.

(A) Boxplots showing relative *CYP2R1* expression in HCC tumor tissues according to modified UICC stage (left; I–IV) and BCLC stage (right; 0, A, B, C/D).

(B) Relative CYP2R1 expression in paired primary tumor (PT) and recurrent tumor (RT) tissues from six representative HCC patients (#P01–#P06), illustrating intra-individual changes at recurrence. Bars indicate mean ± SD. ***p < 0.001. (C) Comparison of *CYP2R1* expression in HCC tumors from patients without (No) or with (Yes) macrovascular invasion. *p < 0.05.

(D) Plasma CYP2R1 protein concentrations (pg/mL) measured by ELISA in normal controls, chronic hepatitis (CH), MASH, cirrhosis, and HCC (Left). *p < 0.05 versus normal. ROC curve evaluating the ability of plasma CYP2R1 to discriminate non-tumor (NT; n = 54) from HCC (n = 88) cases.

Fig. S4

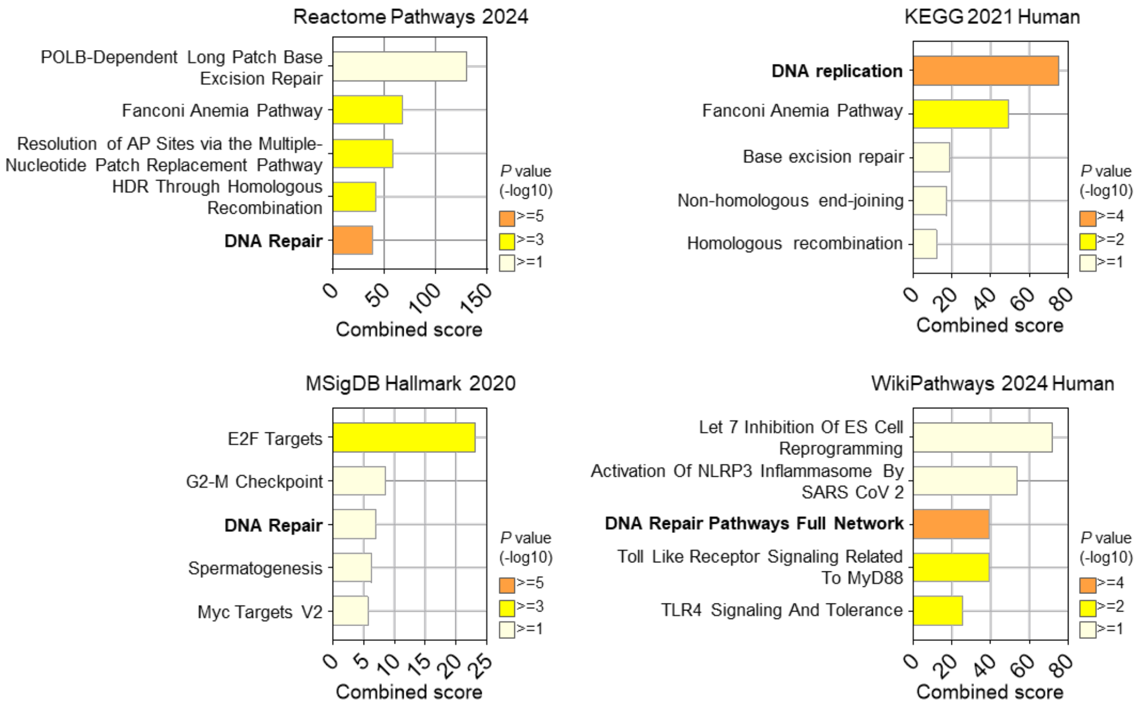

Figure S4. Pathway enrichment profiles associated with the *CYP2R1* co-expression network across four databases.

Bar plots show the top five enriched pathways ranked by combined score in (top left) Reactome Pathways 2024, (top right) KEGG 2021 Human, (bottom left) MSigDB Hallmark 2020, and (bottom right) WikiPathways 2024 Human. Bars represent the combined enrichment score for each term, and colors indicate the nominal p value on a –log10 scale.

Supplementary information

Supplementary Tables

Supplementary Table S1. Pearson correlation coefficients and corresponding p-values for the top 20 differentially expressed CYP genes (10 up-regulated and 10 down-regulated) in the TCGA HCC cohort.

Gene1	Gene2	Correlation	P value	Gene1	Gene2	Correlation	P value
CYP1A2	*CYP3A4*	0.749	6.23e-77	*CYP2B6*	*CYP21A2*	-0.204	2.58e-05
CYP1A2	*CYP2A6*	0.39	9.43e-17	*CYP2B6*	*CYP2R1*	-0.361	2.17e-14
CYP1A2	*CYP2C8*	0.556	1.84e-35	*CYP2B6*	*CYP7A1*	0.262	4.92e-08
CYP1A2	*CYP2E1*	0.542	1.66e-33	*CYP2B6*	*CYP17A1*	-0.059	0.227
CYP1A2	*CYP2B6*	0.493	4.04e-27	*CYP8B1*	*CYP4A11*	0.578	5.82e-39
CYP1A2	*CYP8B1*	0.565	6.95e-37	*CYP8B1*	*CYP2C9*	0.589	1.06e-40
CYP1A2	*CYP4A11*	0.501	3.74e-28	*CYP8B1*	*CYP39A1*	0.488	1.59e-26
CYP1A2	*CYP2C9*	0.559	5.44e-36	*CYP8B1*	*CYP26B1*	-0.459	2.35e-23
CYP1A2	*CYP39A1*	0.652	2.21e-52	*CYP8B1*	*CYP20A1*	-0.232	1.43e-06
CYP1A2	*CYP26B1*	-0.161	0.000902	*CYP8B1*	*CYP27B1*	-0.264	3.72e-08
CYP1A2	*CYP20A1*	-0.182	0.00017	*CYP8B1*	*CYP51A1*	0.073	0.133
CYP1A2	*CYP27B1*	-0.312	5.64e-11	*CYP8B1*	*CYP4F22*	-0.097	0.0464
CYP1A2	*CYP51A1*	0.01	0.833	*CYP8B1*	*CYP1B1*	-0.045	0.357
CYP1A2	*CYP4F22*	-0.292	1.05e-09	*CYP8B1*	*CYP21A2*	-0.13	0.00736
CYP1A2	*CYP1B1*	0.058	0.232	*CYP8B1*	*CYP2R1*	-0.319	2.16e-11
CYP1A2	*CYP21A2*	-0.178	0.000243	*CYP8B1*	*CYP7A1*	0.43	2.47e-20
CYP1A2	*CYP2R1*	-0.355	5.72e-14	*CYP8B1*	*CYP17A1*	0.182	0.000178
CYP1A2	*CYP7A1*	0.06	0.218	*CYP4A11*	*CYP2C9*	0.626	3.44e-47
CYP1A2	*CYP17A1*	-0.085	0.0806	*CYP4A11*	*CYP39A1*	0.515	6.23e-30
CYP3A4	*CYP2A6*	0.494	2.79e-27	*CYP4A11*	*CYP26B1*	-0.32	1.66e-11
CYP3A4	*CYP2C8*	0.632	3.08e-48	*CYP4A11*	*CYP20A1*	-0.203	2.65e-05
CYP3A4	*CYP2E1*	0.477	2.5e-25	*CYP4A11*	*CYP27B1*	-0.326	6.77e-12
CYP3A4	*CYP2B6*	0.506	8.77e-29	*CYP4A11*	*CYP51A1*	0.091	0.0612
CYP3A4	*CYP8B1*	0.696	3.54e-62	*CYP4A11*	*CYP4F22*	0.029	0.552
CYP3A4	*CYP4A11*	0.544	9.59e-34	*CYP4A11*	*CYP1B1*	-0.179	0.000219
CYP3A4	*CYP2C9*	0.619	7.26e-46	*CYP4A11*	*CYP21A2*	-0.199	4.06e-05
CYP3A4	*CYP39A1*	0.54	3.24e-33	*CYP4A11*	*CYP2R1*	-0.381	5.79e-16
CYP3A4	*CYP26B1*	-0.254	1.33e-07	*CYP4A11*	*CYP7A1*	0.266	3.11e-08

CYP3A4	*CYP20A1*	-0.171	0.000428	*CYP4A11*	*CYP17A1*	0.075	0.122
CYP3A4	*CYP27B1*	-0.311	6.41e-11	*CYP2C9*	*CYP39A1*	0.385	2.53e-16
CYP3A4	*CYP51A1*	0.109	0.0253	*CYP2C9*	*CYP26B1*	-0.276	8.38e-09
CYP3A4	*CYP4F22*	-0.264	3.77e-08	*CYP2C9*	*CYP20A1*	-0.21	1.46e-05
CYP3A4	*CYP1B1*	0.032	0.519	*CYP2C9*	*CYP27B1*	-0.278	6.18e-09
CYP3A4	*CYP21A2*	-0.22	5.02e-06	*CYP2C9*	*CYP51A1*	0.062	0.201
CYP3A4	*CYP2R1*	-0.29	1.33e-09	*CYP2C9*	*CYP4F22*	-0.139	0.00429
CYP3A4	*CYP7A1*	0.265	3.37e-08	*CYP2C9*	*CYP1B1*	-0.082	0.0947
CYP3A4	*CYP17A1*	0.039	0.421	*CYP2C9*	*CYP21A2*	-0.094	0.0533
CYP2A6	*CYP2C8*	0.66	6.31e-54	*CYP2C9*	*CYP2R1*	-0.349	1.65e-13
CYP2A6	*CYP2E1*	-0.025	0.613	*CYP2C9*	*CYP7A1*	0.248	2.57e-07
CYP2A6	*CYP2B6*	0.7	3.06e-63	*CYP2C9*	*CYP17A1*	0.093	0.0575
CYP2A6	*CYP8B1*	0.68	1.86e-58	*CYP39A1*	*CYP26B1*	-0.103	0.0354
CYP2A6	*CYP4A11*	0.663	1.21e-54	*CYP39A1*	*CYP20A1*	-0.135	0.00566
CYP2A6	*CYP2C9*	0.475	3.97e-25	*CYP39A1*	*CYP27B1*	-0.224	3.48e-06
CYP2A6	*CYP39A1*	0.475	4.61e-25	*CYP39A1*	*CYP51A1*	0.08	0.101
CYP2A6	*CYP26B1*	-0.337	1.2e-12	*CYP39A1*	*CYP4F22*	-0.033	0.494
CYP2A6	*CYP20A1*	-0.204	2.56e-05	*CYP39A1*	*CYP1B1*	0.032	0.513
CYP2A6	*CYP27B1*	-0.243	4.48e-07	*CYP39A1*	*CYP21A2*	-0.163	0.000777
CYP2A6	*CYP51A1*	0.088	0.0727	*CYP39A1*	*CYP2R1*	-0.268	2.34e-08
CYP2A6	*CYP4F22*	0.179	0.000214	*CYP39A1*	*CYP7A1*	0.092	0.0602
CYP2A6	*CYP1B1*	-0.127	0.009	*CYP39A1*	*CYP17A1*	-0.082	0.0934
CYP2A6	*CYP21A2*	-0.159	0.00106	*CYP26B1*	*CYP20A1*	0.25	1.92e-07
CYP2A6	*CYP2R1*	-0.228	2.35e-06	*CYP26B1*	*CYP27B1*	0.155	0.00143
CYP2A6	*CYP7A1*	0.403	7.32e-18	*CYP26B1*	*CYP51A1*	-0.011	0.819
CYP2A6	*CYP17A1*	0.167	0.000582	*CYP26B1*	*CYP4F22*	0.099	0.0417
CYP2C8	*CYP2E1*	0.201	3.32e-05	*CYP26B1*	*CYP1B1*	0.088	0.0725
CYP2C8	*CYP2B6*	0.598	3.49e-42	*CYP26B1*	*CYP21A2*	0.045	0.354
CYP2C8	*CYP8B1*	0.633	1.48e-48	*CYP26B1*	*CYP2R1*	0.274	1.03e-08
CYP2C8	*CYP4A11*	0.665	4.01e-55	*CYP26B1*	*CYP7A1*	-0.36	2.5e-14
CYP2C8	*CYP2C9*	0.593	2.04e-41	*CYP26B1*	*CYP17A1*	-0.204	2.45e-05
CYP2C8	*CYP39A1*	0.522	9.75e-31	*CYP20A1*	*CYP27B1*	0.177	0.000264
CYP2C8	*CYP26B1*	-0.274	1.11e-08	*CYP20A1*	*CYP51A1*	0.151	0.00183
CYP2C8	*CYP20A1*	-0.199	4.08e-05	*CYP20A1*	*CYP4F22*	0.189	9.35e-05
CYP2C8	*CYP27B1*	-0.207	1.8e-05	*CYP20A1*	*CYP1B1*	0.153	0.00166
CYP2C8	*CYP51A1*	0.071	0.144	*CYP20A1*	*CYP21A2*	-0.144	0.00307
CYP2C8	*CYP4F22*	0.058	0.238	*CYP20A1*	*CYP2R1*	0.366	9.18e-15
CYP2C8	*CYP1B1*	0.013	0.795	*CYP20A1*	*CYP7A1*	-0.025	0.609
CYP2C8	*CYP21A2*	-0.046	0.342	*CYP20A1*	*CYP17A1*	-0.055	0.259
CYP2C8	*CYP2R1*	-0.31	7.98e-11	*CYP27B1*	*CYP51A1*	-0.085	0.0832
CYP2C8	*CYP7A1*	0.227	2.63e-06	*CYP27B1*	*CYP4F22*	0.051	0.294

CYP2C8	*CYP17A1*	0.125	0.0103	*CYP27B1*	*CYP1B1*	0.162	0.000882
CYP2E1	*CYP2B6*	0.194	5.96e-05	*CYP27B1*	*CYP21A2*	0.158	0.00111
CYP2E1	*CYP8B1*	0.3	3.19e-10	*CYP27B1*	*CYP2R1*	0.299	3.95e-10
CYP2E1	*CYP4A11*	0.221	4.69e-06	*CYP27B1*	*CYP7A1*	-0.159	0.00105
CYP2E1	*CYP2C9*	0.39	9.07e-17	*CYP27B1*	*CYP17A1*	0.089	0.0684
CYP2E1	*CYP39A1*	0.262	4.67e-08	*CYP51A1*	*CYP4F22*	0.116	0.0177
CYP2E1	*CYP26B1*	-0.177	0.000257	*CYP51A1*	*CYP1B1*	0.118	0.0154
CYP2E1	*CYP20A1*	-0.15	0.00208	*CYP51A1*	*CYP21A2*	-0.223	3.67e-06
CYP2E1	*CYP27B1*	-0.244	3.95e-07	*CYP51A1*	*CYP2R1*	0.146	0.00264
CYP2E1	*CYP51A1*	-0.057	0.246	*CYP51A1*	*CYP7A1*	0.329	4.52e-12
CYP2E1	*CYP4F22*	-0.302	2.6e-10	*CYP51A1*	*CYP17A1*	0.075	0.126
CYP2E1	*CYP1B1*	-0.059	0.231	*CYP4F22*	*CYP1B1*	0.034	0.49
CYP2E1	*CYP21A2*	-0.067	0.172	*CYP4F22*	*CYP21A2*	0.098	0.0439
CYP2E1	*CYP2R1*	-0.317	2.68e-11	*CYP4F22*	*CYP2R1*	0.155	0.00143
CYP2E1	*CYP7A1*	-0.004	0.929	*CYP4F22*	*CYP7A1*	0.178	0.000232
CYP2E1	*CYP17A1*	-0.07	0.154	*CYP4F22*	*CYP17A1*	0.066	0.177
CYP2B6	*CYP8B1*	0.606	1.28e-43	*CYP1B1*	*CYP21A2*	0.112	0.021
CYP2B6	*CYP4A11*	0.608	7.73e-44	*CYP1B1*	*CYP2R1*	0.193	6.6e-05
CYP2B6	*CYP2C9*	0.475	4.98e-25	*CYP1B1*	*CYP7A1*	0.008	0.876
CYP2B6	*CYP39A1*	0.469	1.86e-24	*CYP1B1*	*CYP17A1*	-0.024	0.621
CYP2B6	*CYP26B1*	-0.271	1.51e-08	*CYP21A2*	*CYP2R1*	0.044	0.369
CYP2B6	*CYP20A1*	-0.174	0.000341	*CYP21A2*	*CYP7A1*	-0.085	0.0808
CYP2B6	*CYP27B1*	-0.226	2.78e-06	*CYP21A2*	*CYP17A1*	0.201	3.12e-05
CYP2B6	*CYP51A1*	0.056	0.255	*CYP2R1*	*CYP7A1*	0.032	0.512
CYP2B6	*CYP4F22*	0.016	0.738	*CYP2R1*	*CYP17A1*	0.084	0.0849
CYP2B6	*CYP1B1*	-0.164	0.000711	*CYP7A1*	*CYP17A1*	0.189	9.43e-05

Supplementary Table S2. Receiver operating characteristic (ROC) analyses of the top 20 differentially expressed CYP genes in TCGA cohort.

	AUC	95% CI	P value
CYP1A2	0.94	0.92 to 0.96	<0.001
CYP3A4	0.84	0.81 to 0.88	<0.001
CYP2A6	0.79	0.75 to 0.83	<0.001
CYP2C8	0.96	0.94 to 0.98	<0.001
CYP2E1	0.79	0.74 to 0.83	<0.001
CYP2B6	0.91	0.88 to 0.94	<0.001
CYP8B1	0.79	0.75 to 0.83	<0.001
CYP4A11	0.94	0.92 to 0.97	<0.001
CYP2C9	0.87	0.84 to 0.91	<0.001
CYP39A1	0.93	0.9 to 0.95	<0.001
CYP26B1	0.56	0.5 to 0.63	0.15
CYP20A1	0.67	0.6 to 0.74	<0.001
CYP27B1	0.85	0.8 to 0.91	<0.001
CYP51A1	0.6	0.53 to 0.67	0.02
CYP4F22	0.55	0.5 to 0.6	0.28
CYP1B1	0.62	0.56 to 0.68	0.005
CYP21A2	0.94	0.92 to 0.96	<0.001
CYP2R1	0.95	0.94 to 0.98	<0.001
CYP7A1	0.65	0.6 to 0.7	<0.001
CYP17A1	0.68	0.63 to 0.73	<0.001

AUC, area under the curve; 95% CI, 95% confidence interval.

Supplementary Table S3. Univariate Cox proportional hazards analysis of clinical variables and CYP gene expression in TCGA HCC cohort.

Variable	coef	HR	P value	Lower 95% CI	Upper 95% CI
Diagnosis Age	0.014	1.014	0.045	1.000	1.028
Sex	-0.216	0.805	0.232	0.565	1.149
Patient Weight	-0.006	0.994	0.219	0.984	1.004
Aneuploidy Score	0.023	1.024	0.031	1.002	1.046
Mutation Count	0.001	1.001	0.024	1.000	1.002
CYP1A2	-0.043	0.958	0.281	0.887	1.035
CYP3A4	-0.059	0.943	0.026	0.896	0.993
CYP2A6	-0.044	0.957	0.102	0.909	1.009
CYP2C8	-0.088	0.915	0.016	0.852	0.984
CYP2E1	-0.044	0.957	0.082	0.911	1.006
CYP2B6	-0.054	0.947	0.243	0.864	1.038
CYP8B1	-0.079	0.924	0.013	0.868	0.983
CYP4A11	-0.092	0.912	0.042	0.836	0.997
CYP2C9	-0.144	0.866	0.000	0.807	0.929
CYP39A1	-0.050	0.951	0.435	0.839	1.078
CYP4A22	-0.087	0.917	0.108	0.825	1.019
CYP2A7	-0.041	0.960	0.274	0.891	1.033
CYP26A1	-0.127	0.880	0.369	0.667	1.163
CYP4F2	-0.101	0.904	0.031	0.824	0.991
CYP1A1	-0.007	0.993	0.866	0.912	1.081
CYP2C19	0.206	1.229	0.172	0.914	1.653
CYP2C18	0.012	1.012	0.830	0.906	1.130
CYP4V2	-0.281	0.755	0.005	0.621	0.918
CYP2J2	-0.013	0.987	0.856	0.858	1.136
CYP3A43	-0.297	0.743	0.012	0.589	0.938
PTGIS	0.051	1.052	0.638	0.851	1.301
CYP3A7	-0.046	0.955	0.229	0.887	1.029
CYP27A1	-0.178	0.837	0.002	0.748	0.937
CYP4F3	-0.125	0.883	0.050	0.779	1.000
CYP4F12	-0.195	0.823	0.020	0.698	0.969
CYP7B1	-0.016	0.984	0.868	0.817	1.186
CYP4X1	-0.017	0.983	0.880	0.789	1.225
TBXAS1	0.278	1.320	0.069	0.978	1.781
CYP3A5	-0.174	0.840	0.002	0.751	0.939
CYP2D6	-0.096	0.909	0.034	0.831	0.993
CYP2A13	-0.021	0.980	0.773	0.852	1.127
CYP2U1	-0.348	0.706	0.077	0.480	1.039
CYP4F11	-0.003	0.997	0.964	0.878	1.133
CYP4Z1	-0.448	0.639	0.371	0.239	1.706
CYP11B1	2.058	7.834	0.034	1.170	52.430
CYP26C1	0.497	1.645	0.874	0.004	751.747
CYP4F8	-1.420	0.242	0.543	0.002	23.403
CYP2F1	5.507	246.484	0.077	0.556	109284.218
CYP46A1	-0.019	0.981	0.981	0.209	4.606
CYP4B1	-0.369	0.692	0.659	0.135	3.554
CYP24A1	0.591	1.806	0.071	0.951	3.430
CYP11B2	0.323	1.382	0.010	1.080	1.768
CYP11A1	-0.203	0.816	0.025	0.683	0.975

CYP2S1	0.181	1.199	0.048	1.001	1.435
CYP27C1	-0.049	0.953	0.890	0.479	1.895
CYP2W1	0.033	1.034	0.906	0.598	1.786
CYP19A1	0.212	1.236	0.069	0.984	1.552
CYP26B1	0.421	1.524	0.000	1.258	1.847
CYP20A1	0.305	1.357	0.292	0.769	2.393
CYP27B1	0.177	1.194	0.386	0.800	1.782
CYP51A1	0.046	1.047	0.657	0.854	1.284
CYP4F22	-0.040	0.960	0.443	0.866	1.065
CYP1B1	-0.022	0.979	0.791	0.834	1.149
CYP21A2	0.126	1.134	0.199	0.936	1.373
CYP2R1	0.329	1.389	0.115	0.923	2.090
CYP7A1	-0.113	0.893	0.005	0.825	0.966
CYP17A1	-0.073	0.930	0.076	0.858	1.008

coef, cox regression coefficient; HR, hazard ratio; Lower 95% CI, lower bound of the 95% confidence interval for HR; Upper 95% CI, upper bound of the 95% confidence interval for HR.

Supplementary Table S4. Multivariate Cox proportional hazards analysis of significant clinical variables and CYP gene expression in TCGA HCC cohort.

Variable	coef	HR	P value	Lower 95% CI	Upper 95% CI
Diagnosis Age	0.011	1.011	0.171	0.995	1.028
Aneuploidy Score	0.016	1.016	0.252	0.989	1.043
Mutation Count	0.002	1.002	0.036	1.000	1.003
CYP3A4	-0.005	0.995	0.914	0.911	1.087
CYP2C8	-0.015	0.985	0.824	0.860	1.128
CYP8B1	0.103	1.108	0.119	0.974	1.261
CYP4A11	0.147	1.159	0.058	0.995	1.349
CYP2C9	-0.133	0.876	0.026	0.779	0.984
CYP4F2	0.050	1.052	0.572	0.883	1.252
CYP4V2	-0.248	0.780	0.099	0.581	1.048
CYP3A43	-0.119	0.888	0.421	0.664	1.187
CYP27A1	-0.180	0.836	0.084	0.681	1.025
CYP4F12	-0.145	0.865	0.176	0.702	1.067
CYP3A5	-0.055	0.947	0.505	0.806	1.112
CYP2D6	0.060	1.062	0.392	0.925	1.218
CYP11B1	-5.115	0.006	0.091	0.000	2.255
CYP11B2	1.003	2.726	0.014	1.224	6.071
CYP11A1	-0.150	0.860	0.159	0.698	1.061
CYP2S1	0.062	1.064	0.585	0.852	1.329
CYP26B1	0.432	1.540	0.001	1.201	1.975
CYP7A1	-0.003	0.997	0.952	0.894	1.111

coef, cox regression coefficient; HR, hazard ratio; Lower 95% CI, lower bound of the 95% confidence interval for HR; Upper 95% CI, upper bound of the 95% confidence interval for HR.

Supplementary Table S5. Correlation of *CYP2R1* expression with eleven canonical MDSC marker genes in the TCGA-LIHC cohort.

	Marker	Partial.rho	*P*
Myeloid-Derived Suppressor Cells (MDSC)	*HLA-DRA*	0.19	3.82e−04
	HLA-DRB1	0.123	2.18e−02
	CD33	0.192	3.35e−04
	CD11b (ITGAM)	0.286	6.48e−08
	CD14 (negative)	0.003	9.52e−01
	IDO (IDO1)	0.12	2.58e−02
	LOX	0.271	3.22e−07
	S100A8	0.074	1.71e−01
	S100A9	0.149	5.65e−03
	CD80	0.238	8.08e−06
	CD83	0.348	3.12e−11

Supplementary Table S6. Baseline clinicopathological features of the tissue cohort.

Variables	n = 110
Age (years), mean ± SD	55.78 ± 10.08
Male sex, n (%)	86 (78.2)
Etiology, n (%)	
HBV	103 (93.6)
HCV	4 (3.6)
Alcohol	2 (1.8)
HCV+Alcohol	1 (0.9)
BMI (kg/m^2), mean ± SD	24.12 ± 3.70
Smoking, n (%)	40 (36.4)
Alcohol Consumption, n (%)	38 (34.9)
Cirrhosis, n (%)	30 (27.3)
AST (IU/L), mean ± SD	43 ± 55
ALT (IU/L), mean ± SD	40 ± 40
Platelet (x10^3/µL), mean ± SD	175.52 ± 70.43
AFP (ng/mL), mean ± SD	2382.80 ± 7622.61
PIVKA-II (mAU/mL), mean ± SD	5236.25 ± 14725.34
Albumin (g/dL), mean ± SD	4.43 ± 0.57
Total bilirubin (mg/dL), mean ± SD	0.81 ± 1.26
Creatinine (mg/dL), mean ± SD	1.04 ± 1.45
Sodium (mmol/L), mean ± SD	139.35 ± 2.13
Macrovascular invasion, n (%)	38 (34.5)
Microvascular invasion, n (%)	61 (55.5)
Lymph node metastasis, n (%)	7 (6.4)
Distant metastasis, n (%)	23 (20.9)
Modified UICC stage, n (%)	
I	24 (21.8)
II	45 (40.9)
III	27 (24.5)
IVA	10 (9.1)
IVB	4 (3.6)
BCLC stage, n (%)	
0	24 (24.8)
A	61 (55.5)
B	10 (9.1)
C	12 (10.9)
D	3 (2.7)
Recurrence, n (%)	10 (9.1)

HBV, Hepatitis B Virus; HCV, Hepatitis C Virus; BMI, Body mass index; AST, Aspartate aminotransferase; ALT, Alanine aminotransferase; AFP, α-fetoprotein; PIVKA-II, prothrombin-induced by vitamin K absence or antagonist-II; UICC, Union for International Cancer Control.; BCLC, Barcelona Clinic Liver Cancer.
